# Genetic diversity and kelp forest vulnerability to climatic stress

**DOI:** 10.1038/s41598-018-20009-9

**Published:** 2018-01-30

**Authors:** Thomas Wernberg, Melinda A. Coleman, Scott Bennett, Mads S. Thomsen, Fernando Tuya, Brendan P. Kelaher

**Affiliations:** 1 UWA Oceans Institute (M470) and School of Biological Sciences, University of Western Australia, Crawley, 6009 WA Australia; 2 Department of Primary Industries, NSW Fisheries, PO Box 4321, Coffs Harbour, NSW 2450 Australia; 3 National Marine Science Centre & Centre for Coastal Biogeochemistry Research, School of Environment, Science and Engineering, Southern Cross University, PO Box 4321, Coffs Harbour, NSW 2450 Australia; 4 Department of Global Change Research, Institut Mediterrani d’Estudis Avançats (Universitat de les Illes Balears-Consejo Superior de Investigaciones Científicas), Esporles, Spain; 5 Marine Ecology Research Group and Centre for Integrative Ecology, School of Biological Sciences, University of Canterbury, Private Bag 4800 Christchurch, New Zealand; 6 IU-ECOAQUA, Universidad de Las Palmas de Gran Canaria, 35017 Las Palmas, Canary Islands Spain

## Abstract

Genetic diversity confers adaptive capacity to populations under changing conditions but its role in mediating impacts of climate change remains unresolved for most ecosystems. This lack of knowledge is particularly acute for foundation species, where impacts may cascade throughout entire ecosystems. We combined population genetics with eco-physiological and ecological field experiments to explore relationships among latitudinal patterns in genetic diversity, physiology and resilience of a kelp ecosystem to climate stress. A subsequent ‘natural experiment’ illustrated the possible influence of latitudinal patterns of genetic diversity on ecosystem vulnerability to an extreme climatic perturbation (marine heatwave). There were strong relationships between physiological versatility, ecological resilience and genetic diversity of kelp forests across latitudes, and genetic diversity consistently outperformed other explanatory variables in contributing to the response of kelp forests to the marine heatwave. Population performance and vulnerability to a severe climatic event were thus strongly related to latitudinal patterns in genetic diversity, with the heatwave extirpating forests with low genetic diversity. Where foundation species control ecological structure and function, impacts of climatic stress can cascade through the ecosystem and, consequently, genetic diversity could contribute to ecosystem vulnerability to climate change.

## Introduction

A core tenet of evolutionary theory is that the ability of species to adapt and persist through changing environments is contingent on latent functional responses suited to new conditions^1^. Genetic variation among individuals within a population provides a mechanistic basis for plasticity and adaptability, such that a multiplicity of genotypes (genetic diversity) provides a greater range of possible functional responses (physiological versatility), and thus a higher probability that a population will resist, or recover from, a perturbation (ecological resilience)^2,3^. Despite an advanced conceptual understanding of the implied positive relationships among genetic diversity, physiological versatility and ecological resilience, empirical evidence for their existence is lacking from natural populations, and strongly biased towards experiments on model organisms and clonal plants^4^. Knowledge about the role of genetic diversity in underpinning species performance and ecosystem vulnerability is, however, critical to successfully mitigate impacts of pressures, such as population over-exploitation, pollution, invasive species and global warming.

Increasing temperatures have already impacted most ecosystems on the planet^5–7^. The mechanisms that translate the physical forcing of climate change into biological changes are, however, poorly understood, creating major uncertainty about future ecological scenarios^8,9^, and limiting our ability to predict impacts^9^ and implement mitigation strategies, such as targeted conservation and rehabilitation^10,11^. A critical knowledge gap relates to the response of species to impending changes, and the role of genetic factors in mediating population persistence through latent functional responses^4,9,12^. That is, the resilience of a population to climatic stress might depend on possessing sufficient genetic variation to allow a range of responses to stressors, some of which will promote population persistence.

Our lack of understanding of how genetic diversity mediates population response to climate stress is particularly acute for ‘foundation species’ (trees, corals, kelps, etc.)^4,12^, because of their critical influence on community organization and ecosystem functioning. Indeed, the importance of foundation species in mediating climate stress for associated biodiversity is likely to increase in a warmer future^2,13^. Where populations of foundation species have reduced potential to respond to environmental stress, resilience might be compromised^14^ and, if perturbations are severe, entire populations could perish with impacts cascading through the ecosystem^15,16^. Knowledge of the role of genetic diversity in determining the response of foundation species to climatic stress is, therefore, particularly critical for assessing the overall vulnerability of an ecosystem, and to ensure successful conservation and management strategies^2,4,11,13,17,18^.

Here, we combined population genetics^19^ with eco-physiological and ecological field experiments^14^ to examine relationships between latitudinal patterns in genetic diversity, physiological versatility and ecological resilience of kelp forests, one of the most important foundation species of temperate marine habitats globally^20,21^. We also documented subsequent responses of kelp (*Ecklonia radiata*) forests to an extreme climatic perturbation (a marine heatwave), thus demonstrating through a ‘natural experiment’, the possible influence of latitudinal patterns in genetic diversity on ecosystem vulnerability to climatic stress.

## Results

**Figure 1:**
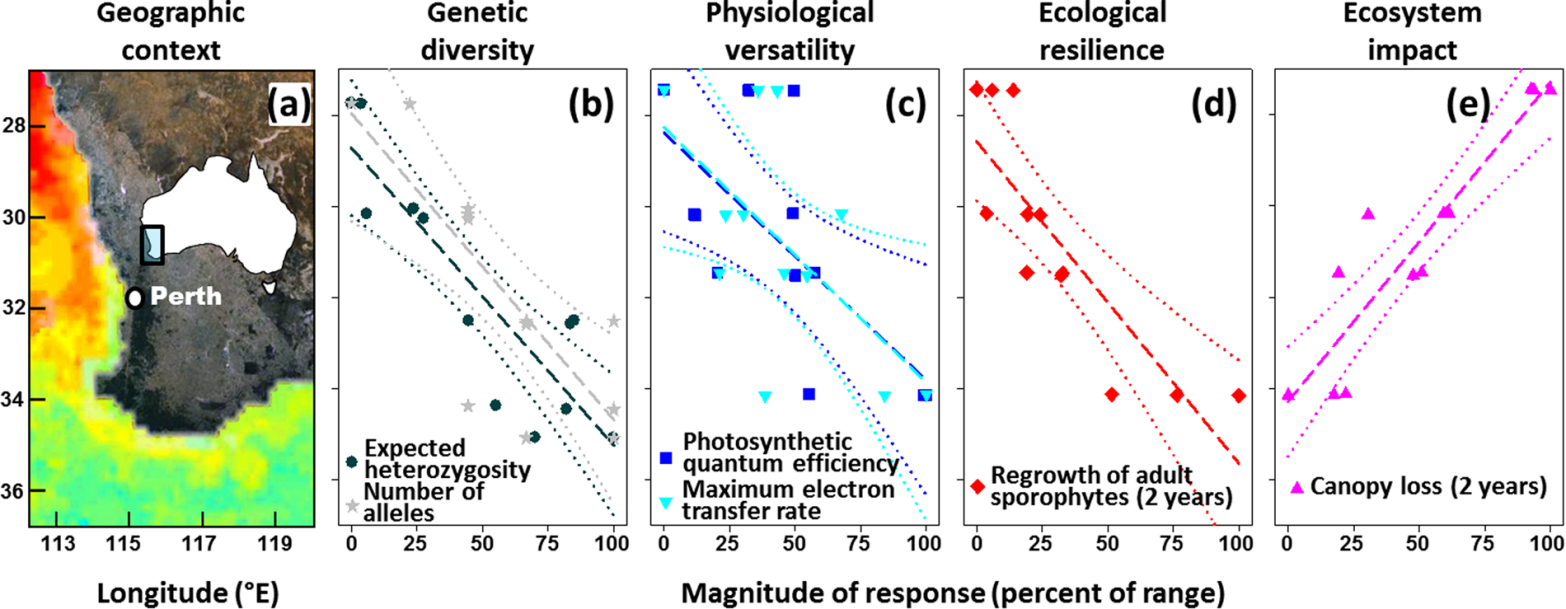
Geographic context and population characteristics for 12 Australian kelp forests. (**a**) The coastline of southwestern Australia is swept by the poleward flow of the warm Leeuwin Current, which creates a uniform gradient in ocean temperature of 2–3 °C across latitudes from 27°S to 35°S (annual daily mean 21.9 to 19.5 °C, respectively) (Wernberg & Smale, 2009, see also Fig. S1). Prior to the 2011 marine heat wave, kelp forests had their equatorward limit in Kalbarri (27.7°S). (**b**) Genetic diversity (data range: H_e_ 0.269–0.375, N_a_ 12–21)^19^, (**c**) physiological versatility (data range: α 2.8–12.4%, ETR_max_ 2.3–22.2%) and (**d**) ecological resilience (data range: 1.2–11.1 kelps m^−2^)^14^ of kelp forests measured prior to the heat wave. (**e**) Ecosystem impact (data range: −86–26% change in kelp forest cover) of the 2011-extreme heat wave measured two years after the event. Latitude is shown on the y-axis for all panels and scaled population characteristics on the x-axis for panels (b–e). Lines represent linear regressions (dashed) with associated 95% confidence limits (dotted). Regression coefficients are given in Table S1. The map (Fig. 1a) was generated in Google Earth version 7.1.8.3036 (https://www.google.com/earth/; © CNES/SpotImage, Data SIO, NOAA, US Navy, NGA, GEBCO) and modified using GIMP version 2.8.14 (https://www.gimp.org/). This included drawing and adding the insert map of Australia.

We measured genetic diversity, physiological versatility and ecological resilience in 12 kelp forests (*Ecklonia radiata*) along a latitudinal gradient in Western Australia^14^ (Fig. 1a). *E. radiata* is a dominant foundation species on reefs throughout temperate Australasia^21^, where it exerts a critical influence on biodiversity and ecosystem functioning^22,23^.

We found a strong, positive, relationship between latitude and genetic diversity of kelp, measured as both expected heterozygosity (r^2^ = 0.74, P = 0.002) and number of alleles (r^2^ = 0.58, P = 0.007), across southwestern Australia (Fig. 1b, see appendix S1 for additional regression statistics). We also found significant, positive, relationships between latitude and physiological versatility (quantum efficiency: r^2^ = 0.44, P = 0.037; maximum electron transfer rate: r^2^ = 0.36, P = 0.041; Fig. 1c) and ecological resilience (r^2^ = 0.68, P = 0.009, Fig. 1d). That is, compared to kelp forests at high (cool) latitudes, kelp forests at low (warm) latitudes had less genetic variation, a narrower range of physiological responses to changes in canopy cover, and suppressed regrowth following experimental canopy loss.

**Figure 2:**
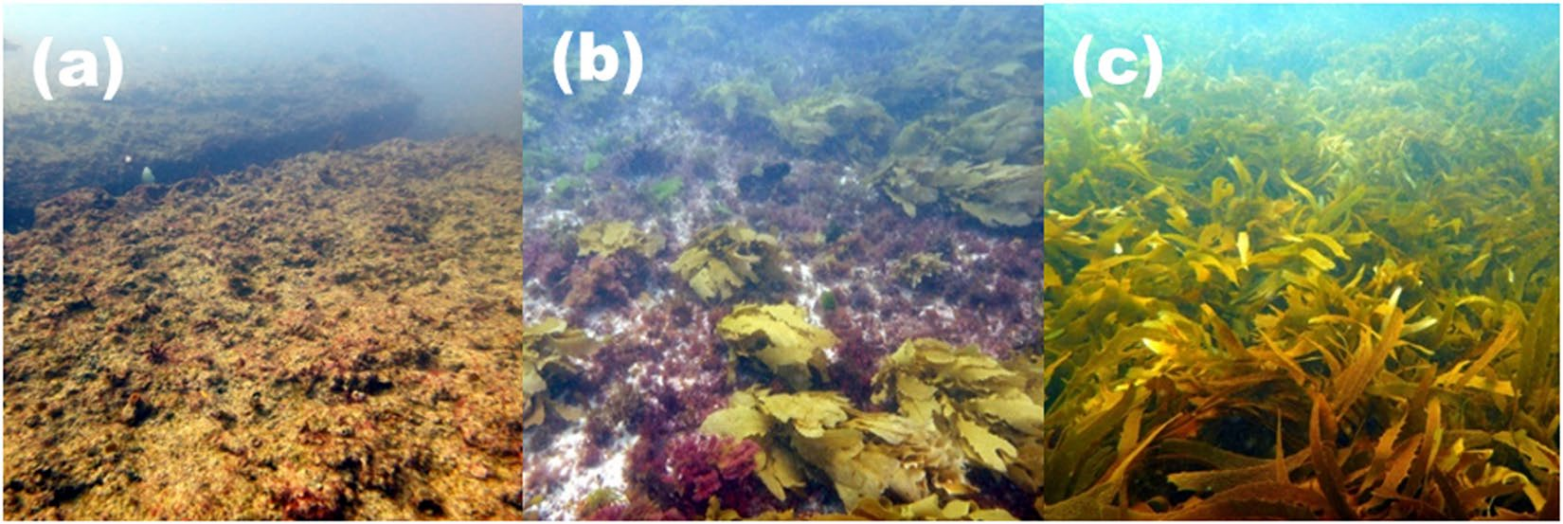
Impact of the 2011 marine heat wave on kelp forests with different genetic diversities. During the Austral summer of 2011, an extreme marine heat wave devastated low latitude kelp forests with low genetic diversity (**a**), whereas forests with intermediate genetic diversity showed partial kelp canopy loss (**b**) and high latitude high-diversity forests showed no discernible impact on kelp canopy cover (**c**) despite similar temperature anomalies (Fig. S2). Prior to the 2011 marine heat wave, there were no differences in kelp canopy cover among these kelp forests^14^. All photos taken by T. Wernberg.

Subsequently, these 12 kelp forests experienced an extreme marine heatwave, where ocean temperatures along 2,000 km of coastline soared above anything seen for at least 140 years^24,25^. The latitudinal patterns of genetic diversity, physiological versatility, and ecological resilience were mirrored in kelp forest response to the heatwave (r^2^ = 0.84, P < 0.001; Fig. 1e), with strikingly different impacts observed across the 12 kelp forests (Figs 1e, 2). Despite all forests experiencing similar monthly climatological maximum anomalies during the five months capturing the heatwave (appendix S2), suggesting a similar magnitude of deviation from the norm, low latitude forests with the lowest genetic diversity disappeared completely (Fig. 2a), whereas there were no discernible changes in high latitude forests with the highest genetic diversity (Fig. 2c). Kelp forests at mid latitudes showed partial canopy loss (Fig. 2b).

To investigate possible drivers of these patterns, we compared the contribution of several potential physical and biological predictors (Table 1, appendix S3). Genetic diversity was the only one to consistently rank as the top predictor of heatwave impact (Table 1) being selected in all of the five top performing (lowest AICc) combinations of predictors (Table 1). In contrast, the heatwave alone (measured as cumulative monthly climatological maximum anomalies ~ sum of temperatures exceeding the long-term maximum for each region^26^), explained little variation in impact and was only selected (with genetic diversity) in one of the five top performing combinations of predictors (Table 1).

**Table 1:** Distance-based linear modelling relating physical and biological predictors (appendix S3) of kelp forest responses to the heatwave.

Marginal tests				
Predictor variable	**SS(trace)**	**Pseudo-F**	**P**	**Variation explained**
Genetic diversity (H_e_)	13334	58.00	0.0002	85.3%
Heatwave	4732	4.34	0.07	30.2%
Nutrient concentration	4059	3.51	0.09	26.0%
Reef topography	1607	1.15	0.31	10.3%
Turf/foliose seaweeds	1090	0.75	0.40	7.0%
Wave exposure	788	0.53	0.48	5.0%
Fish herbivores	165	0.11	0.76	1.1%
Depth	43	0.03	0.87	0.3%
**Best model selection**				
**AICc**	**r** ^**2**^	**Predictor variables**		
68.4	0.85	Genetic diversity		
69.6	0.88	Genetic diversity, Reef topography		
70.3	0.87	Genetic diversity, Heatwave		
70.9	0.87	Genetic diversity, Nutrient concentration		
71.0	0.87	Genetic diversity, Turf/foliose seaweeds		

Top half: Marginal tests ascertaining the relationships to individual predictors (total trace = 15633). Bottom half: Multiple regression to ascertain the best (lowest AICc) combinations of predictors, showing the five best models overall.

## Discussion

Current understanding of how genetic diversity mediates ecosystem vulnerability to climatic stress has a strong theoretical basis, but evidence from natural populations is rare. Here, we provided empirical evidence for strong covariation between functional responses and population vulnerability to environmental stress, and latitudinal patterns in genetic diversity of kelp. These insights were only possible through a unique combination of independent broad scale field studies, controlled manipulations, and a ‘natural disturbance experiment’ affecting an entire coastline. Given the opportunistic and correlative nature of events and data collections, our study was not designed to tease apart the exact causal mechanisms linking latitudinal patterns of genetic variation, physiological and ecological performance, and population impact. Moreover, identifying underlying causes of these patterns is challenging, because latitudinal gradients often integrate multiple mechanisms, many of which are not mutually exclusive, and can covary themselves. However, even if significant association between predictors and responses do not prove cause-effect relationships *per se* (e.g.^27^), such observations remain critical as a foundation for subsequent detailed experiments to pin-point processes. Moreover, because other studies have found limited outcrossing to strongly reduce kelp sporophyte fecundity and fitness^28^, and linked kelp survival and regrowth after canopy loss to variation in photo-physiology^29,30^, there exists a plausible mechanistic link between genetic diversity, physiological performance and canopy persistence in the face of disturbance. This link should underpin future studies designed to definitively demonstrate causality.

Kelp forests are generally robust to disturbances, as canopies can recover through recruitment of new gametophytes^31^, from surviving ‘seed banks’ of microscopic stages that can persist for many months under unfavorable conditions^32^, or from macroscopic sporophytes resisting the perturbation^29^. Nevertheless, our experiments prior to the heatwave revealed systematic differences in the physiology and capacity for recovery and regrowth across the 12 kelp forests, matching the latitudinal patterns of genetic diversity. This is consistent with the hypothesis that the initially observed differences in impact of the heatwave were not caused by an external disturbance alone (e.g., the heatwave itself). Nevertheless, subsequent trajectories of recovery could have been influenced by post-disturbance forces such as Allee effects^33^, or concurrent changes in consumer pressure^34^. Almost six years have now elapsed without any signs of recovery in the most heavily impacted populations^25^. No macroscopic sporophytes have been observed (Wernberg & Bennett, personal observation). Because ‘seedbank’ longevity is thought to be less than one year^32^, and new zoospores would have to arrive against flow of the predominant current^15^, it seems unlikely these kelp forests will recover in the near future.

Several mechanisms could explain the differences in genetic diversity among kelp forests along the latitudinal gradient and have implications for interpreting the response of kelp forests to the heatwave. These include (i) reduced connectivity or smaller effective population sizes in marginal populations^19,35–38^, (ii) geometric constraints on the distribution of genotypes (a ‘mid-domain effect’^39^), (iii) selection for a narrow subset of stress-tolerant genotypes at low latitudes^35,40,41^, or (iv) historical extinction or colonization events^42,43^. These mechanisms are not mutually exclusive and might occur simultaneously. For example, selection for stress tolerant genotypes and restricted connectivity are both likely to be important where a population is contracting from deteriorating environmental conditions at its range edge (e.g., shifting isotherms^35,41^). Teasing apart the mechanism behind the observed patterns of genetic diversity is beyond the scope and capacity of the present study, however, given that the response of low latitude kelp forests to the marine heatwave (extirpation) was opposite to what one would expect for stress-tolerant genotypes (persistence), it is unlikely that selection was the primary mechanism driving the observed patterns of genetic diversity^44^.

We propose that latitudinal variation in genetic diversity may have played a role in mediating the response of kelp forests to the marine heatwave. First, high genetic diversity coincided with slightly more benign (cooler) pre-heatwave conditions, possibly protecting these kelp forests against change (Fig. S4c, “H”, open circle), whereas low diversity forests experienced warmer initial conditions, predisposing them to an abrupt population crash following the heat wave (Fig. S4c, “K”, open circle). Thus, despite both pre-heatwave temperature and genetic diversity covarying among populations and likely being important in determining kelp response, differences among initial temperature conditions alone cannot fully explain the observed impact to kelp forests. The partial mortality of populations within six of the observed sites – characterized by intermediate genetic diversity – highlights the high variability in stress tolerance among individual kelp plants. If within-population genetic differences in stress tolerance did not play a role in driving the observed impacts, then a more pronounced threshold response to the heatwave would have been expected in all kelp forests, irrespective of genetic diversity, with very low and high impacts, below and above the temperature threshold, respectively. The differences in population persistence following the heatwave suggest that while factors such as initial temperature conditions and absolute magnitude of the anomaly might have contributed to the observed impacts (Fig. S4c,f), variability in stress tolerance among different genotypes, as well as the proportion of genotypes in each population, are likely to have contributed to the observed responses.

We suggest that low diversity populations represent a subset of genotypes created via processes such as low connectivity and effective population sizes^36^ rather than prior selection for thermal stress (Fig. S4), where these populations would instead represent a subset of genotypes with higher tolerance to warmer ambient conditions. If the latter had been the case, low diversity, but better adapted populations, would have had higher persistence during the heat wave; this was contrary to the observed changes. We cannot rule out the possibility of some local thermal adaptation within populations, as has been observed in a range of organisms (e.g.^45,46^). Indeed, in the wake of the population impacts observed here, it is conceivable that genotypic frequencies, or population tolerances^46^, now reflect selection, with only tolerant genotypes surviving (but see Pearson *et al*.^44^). Although this study cannot establish causal links it strongly suggests that genetic diversity may at least partially explain the dramatic differences in observed population impacts.

The relationships among genetic, physiological, and ecological population-level responses of kelp forests occurred over a temperature gradient of similar magnitude to projected sea temperature increases in the upcoming 50–100 years^10^. It is therefore plausible that impending environmental changes will cause substantial ecological changes in temperate marine ecosystems, and that patterns of genetic diversity could play a role in mediating the manifestations of these impacts. Importantly, our data provides an empirical example of how functional responses and vulnerability to environmental stress might be anticipated by mapping population level genetic diversity. This result forms the basis for future work establishing general genetic diversity relationships that are independent of latitude and may transcend geographic settings and stressors. Establishing such predictive relationships of genetic diversity for conferring physiological versatility and ecological resilience is particularly critical for foundation species, because they exert an essential influence on biodiversity and energy flow across multiple trophic levels^2,4,13^. Moreover, establishing such relationships is vital to assessing the vulnerability of populations^9^, especially where genetic diversity might already be low^4,35^ or exclusively maintained in refugia^38,47,48^, where future exploitation^37^ or climatic forcing is predicted to be greatest^49^ and where normal mechanisms of recovery might be compromised (e.g., limited propagule supply or invasion of consumers^15,17,50,51^). Loss of genetic diversity could drive widespread loss of physiological versatility and ecological resilience, with flow-on effects cascading through to critical, and potentially irreversible, changes to ecosystem structure and functioning.

## Methods

We examined potential drivers of ecosystem vulnerability to climate stress by comparing baseline datasets that compared latitudinal patterns in genetic diversity, physiology and resilience, to the response of kelp forest ecosystems to a natural climatic event. *Ecklonia radiata* is the only true Laminarian kelp along this coastline so the term “kelp” refers to *Ecklonia radiata* throughout this manuscript. However, *Ecklonia radiata* forms both monospecific^52^ and mixed forests with fucoids^53^ and here we sampled monospecific forests or, for canopy removal experiments, those comprised of at least 50% *Ecklonia radiata* cover.

Baseline data on physiological versatility and ecological resilience, as well as subsequent population persistence following an extreme climatic perturbation (a marine heatwave), were measured in 12 kelp forests (*Ecklonia radiata*) along a latitudinal gradient in Western Australia^14^, Fig. 1a. We sampled three kelp forests (>1 km apart) within each of four regions (>250 km apart), extending ~7° latitude poleward from the warm range-edge of kelps in southwestern Australia (Fig. 1a). Physiological versatility was measured in 2006 and ecological resilience between 2006–2008^14^. The response of the same kelp forests to the heatwave which occurred in 2011^26^, was measured after the event in 2013^54^. Baseline genetic diversity was estimated using kelp plants collected along the same latitudinal gradient and locations, but slightly different sites (a few km apart) in 2006^19^. Genetic diversity was thus matched to each exact kelp forest using its strong relationship with latitude (Table S2). Given the patterns of isolation by distance along this coastline^19^ this approach is justified.

Genetic diversity was measured as expected heterozygosity (H_e_) and number of alleles (N_a_), two of the most commonly reported diversity metrics e.g.^4^, using 6 proven microsatellite markers, in 12 populations, 32 kelps per population^19,55^. Although these are neutral markers that do not enable firm inferences about the effects of genetic diversity on selection or adaptation, there is evidence that neutral marker diversity can be positively correlated with key demographic parameters of population performance, such as reproductive success^56^ and survival of juveniles and adults^57–59^. Importantly, these markers can demonstrate key attributes of breeding systems, such as gene flow and the level of population isolation, factors which correlate with fitness and evolutionary potential^60^. Indeed, in Western Australia *E. radiata* populations are characterized by strong isolation by distance driven by poleward flowing boundary currents^19,61,62^.

Physiological versatility was measured as the coefficient of variation (standard error divided by the mean) in photosynthetic performance (see below for details) of kelp recruits (8–10 kelps per population) 80 days after experimental kelp canopy removal^14^. The coefficient of variation was used because it provides an estimate of variation in responses within each population, unbiased by potential absolute differences in light climate or physiological performance, and thus links with our hypothesis that greater genetic diversity confers a greater range of responses to stress. The canopy removal treatment mimicked localized canopy loss, a stressor which is predicted to escalate as climate change drives increasingly severe heatwaves, storms, and shifts the distribution and abundance of major herbivores^10,50^. Photosynthetic quantum efficiency (α) and maximum electron transfer rate (ETRmax) of photosystem two (PSII) were measured by Pulse Amplitude Modulated [PAM] fluorometry after 15 minutes of dark-adaption^14^. While these measurements were instantaneous, the treatment responses integrated the capacity for long-term (80 days) acclimation to canopy loss. Photosynthesis is a fundamental metabolic process in seaweeds and variation in photosynthetic performance, including quantum efficiency, has been linked to differences in kelp survival and kelp canopy recovery in the face of perturbation, presumably as a consequence of differences in tolerance to excessive light and photoinhibition^29,63^. Moreover, photosynthetic performance has been directly linked to climatic perturbation in both juvenile^14,64^ and adult^65,66^ *E. radiata*.

Ecological resilience of kelp forests was measured as kelp canopy re-growth two years after experimental kelp canopy removal (mean density of re-established adult kelps in six plots of complete canopy removal per population^14^). Our measurements of kelp canopy re-growth do not differentiate between micro- and macroscopic kelp recruits surviving in the understory, and new recruitment from surrounding adults. This measure of resilience, therefore, integrates elements of both resistance to, and recovery from, disturbances.

Population impacts in response to an extreme natural climatic perturbation – a marine heatwave (appendix S2) - were assessed from changes in landscape-scale cover of kelp forests surveyed in 2005 (prior to the heatwave), and again in 2013 (after the heatwave). For each kelp forest, the canopy cover was determined along ten haphazardly positioned, non-overlapping, 25 m transects, first in 2005^52^ and then again in 2013. Regular visits to all kelp forests between 2005 and December 2010 (the onset of the event) showed no visible changes to kelp cover in the period prior to the heatwave^67^, T. Wernberg & S. Bennett personal observation.

To allow better direct visual comparisons of all response variables across latitudes, the magnitude of response in each population characteristic was scaled as a percentage of the range across all 12 kelp forests. However, the range of raw values for each population characteristic are given in the Fig. 1 legend. The strength of relationships between latitude and genetic diversity (H_e_ and N_a_), physiological versatility, resilience and heatwave impact were tested with linear mixed models using R v.3.2.2. For these analyses, the *nlme* package was used to model data with ‘Region’ included as a random factor to account for underlying spatial autocorrelation among sites (n = 12). The r^2^ from the linear mixed model was calculated using the *sem.model.fits* function based on the marginal r^2^ formula of Nakagawa and Schielzeth^68^. This marginal r^2^ quantifies the proportion of total variability that is explained by the fixed effects terms in the model.

Distance-based linear modeling and redundancy analysis was used to identify the relative strength of relationships between population impacts following the heatwave, and seven potential physical and biological predictor variables (appendix S3). Because genetic diversity (Fig. 1b) was sampled independently of the other population characteristics i.e. at slightly different sites (Fig. 1c-e), genetic diversity was attributed to each exact kelp forest using its strong relationship with latitude (Table S2). Thus, it was not possible to include latitude as a predictor in the models because of its correlation to genetic diversity. More importantly, however, latitude does not carry any mechanistic process in itself but is a proxy for processes that may vary along the same scales^69^. Instead of using a proxy, we used a range of measured site characteristics that are widely known to influence kelp forests (e.g. nutrients, depth, herbivory, wave exposure, reef topography, cf. Table S2). Given H_e_ and N_a_ covary across latitude we chose to use only H_e_ as a measure of genetic diversity in these analyses. Based on geometric (Euclidian) distances between samples, these analyses are analogous to standard multiple regressions^70^, where significance is obtained through a randomization test (here 9999 permutations of residuals under a reduced model), thus avoiding assumptions about normal distribution of data and residuals (for technical details, please refer to^70^). To identify which predictor combinations best explained variation in kelp forest responses to the heatwave, we used a model selection procedure with an exhaustive search among all predictor combinations and a distance-based analogue to the Akaike’s Information Criterion, modified to accommodate small sample sizes relative to the number of predictor variables (AICc). This procedure identified the most parsimonious (lowest AICc) predictor subsets see^70^, for, technical, details, while also taking co-linearity among predictors (*r*^2^ < 0.42, Fig. S3) into account^71^. To be conservative, we interpreted these analyses qualitatively because 12 sites (albeit large replication in a field setting) provides low power to quantitatively partition variation among sets of multiple predictor variables.

## Electronic supplementary material

Supplementary Information
