# Supplementary material for: Genetic diversity and kelp forest vulnerability to climatic stress
Source: Sci Rep. 2018 Jan 30;8:1851. doi: 10.1038/s41598-018-20009-9 (PMC5790012; doi:10.1038/s41598-018-20009-9)
Supplement: Supplementary file 1 — Supplementary Information [file 41598_2018_20009_MOESM1_ESM.pdf]

SUPPORTING INFORMATION

Genetic diversity and kelp forest vulnerability to climatic stress

Thomas Wernberg[1,†,*], Melinda A. Coleman[2,3,†], Scott Bennett[1,4], Mads S. Thomsen[1,5], Fernando Tuya[6], Brendan P. Kelaher[3]

[1] UWA Oceans Institute (M470) and School of Biological Sciences, University of Western Australia, Crawley 6009 WA, Australia. Thomas.wernberg@uwa.edu.au

[2] Department of Primary Industries, NSW Fisheries, PO Box 4321, Coffs Harbour NSW 2450, Australia. Melinda.coleman@gmail.com

[3] National Marine Science Centre & Centre for Coastal Biogeochemistry Research, School of Environment, Science and Engineering, Southern Cross University, PO Box 4321, Coffs Harbour NSW 2450, Australia. Brendan.kelaher@scu.edu.au

[4] Department of Global Change Research, Institut Mediterrani d'Estudis Avançats (Universitat de les Illes Balears - Consejo Superior de Investigaciones Científicas), Esporles, Spain. Sbennett@imedea.uib-csic.es

[5] Marine Ecology Research Group and Centre for Integrative Ecology, School of Biological Sciences, University of Canterbury, Private Bag 4800, Christchurch, New Zealand. Mads.solgaard.thomsen@gmail.com

[6] IU-ECOAQUA, Universidad de Las Palmas de Gran Canaria, 35017 Las Palmas, Canary Islands, Spain. Ftuya@yahoo.es

[†] Shared lead authorship.

[*] **Correspondence:** Thomas Wernberg, UWA Oceans Institute (M470) and School of Biological Sciences, University of Western Australia, Crawley 6009 WA, Australia. thomas.wernberg@uwa.edu.au, Ph. +61 8 6488 7204.

Appendix S1: Statistical results from GLMM

Appendix S2: Ocean temperatures during the 2011 marine heat wave

Appendix S3: Physical and biological predictor variables

Appendix S4 Conceptual models

Appendix S5 Additional references

Appendix S1: Statistical results from GLMM

Table S1. Results of linear mixed modelling of the relationships between latitude and genetic diversity (expected heterozygosity and number of alleles), physiological versatility (CV of quantum efficiency and maximum electron transfer rate), resilience and heat wave impact. Each analysis included 'Region' as a random effect to account for any spatial correlation among sites within regions. Analyses were undertaken in *nlme* package using R v.3.2.2. r^2 values were calculated using the *sem.model.fits* function based on the formulas of Nakagawa and Schielzeth (2013).

**Fixed effect**	**Marginal r^2**	**Conditional r^2**	**t**	**P**
Expected heterozogosity	0.742	0.806	4.647	0.002
Number of alleles	0.583	0.599	3.783	0.007
Quantum efficiency	0.438	0.524	2.557	0.037
Maximum electron transfer rate	0.363	0.363	2.505	0.041
Resilience	0.676	0.803	3.586	0.009
HW impact	0.841	0.841	7.616	<0.001

Appendix S2: Ocean temperatures during the 2011 marine heat wave

Temperature conditions experienced by the 12 kelp forests were derived from blended SST data provided by the National Weather Service and the NOAA Operational Model Archive Distribution Systems (NOMADS) (Wernberg *et al.* 2013). Monthly mean sea surface temperatures, and temperatures for the climatological period 1981-2010, were extracted for four regions encompassing the 12 kelp forests from Kalbarri (27.5°S 113.5°E to 28.5°S 114.5°E), Jurien Bay (29.5°S 114.5°E to 30.5°S 115.5°E), Perth (31.5°S 115.5°E to 32.5°S 116.5°E) and Hamelin Bay (33.5°S 114.5°E to 34.5°S 115.5°E). These SST's closely reflect temperatures at 8 - 12 m depth on the reefs where the kelps are found (Smale & Wernberg 2009). Moreover, bottom temperatures vary <5% between individual reefs within our regions (Smale & Wernberg 2009).

In early 2011 (austral summer), the west coast of Australia experienced an extreme marine heat wave driven by an exceptionally strong flow of the Leeuwin Current and high air-sea heat fluxes (Feng *et al.* 2013; Wernberg *et al.* 2013). The marine heat wave resulted in ocean temperatures warmer than anything in 140-years of records from the Hadley Centre (Wernberg *et al.* 2013) and 215 years of records from local coral cores (Zinke *et al.* 2014). In the years immediately prior to the heat wave, summer temperatures were within the warmest temperatures generally experienced (i.e., below the maximum climatological value), but during the heat wave, temperate reefs experienced extended periods of temperatures 2-3 °C higher than normal (Fig. S1).

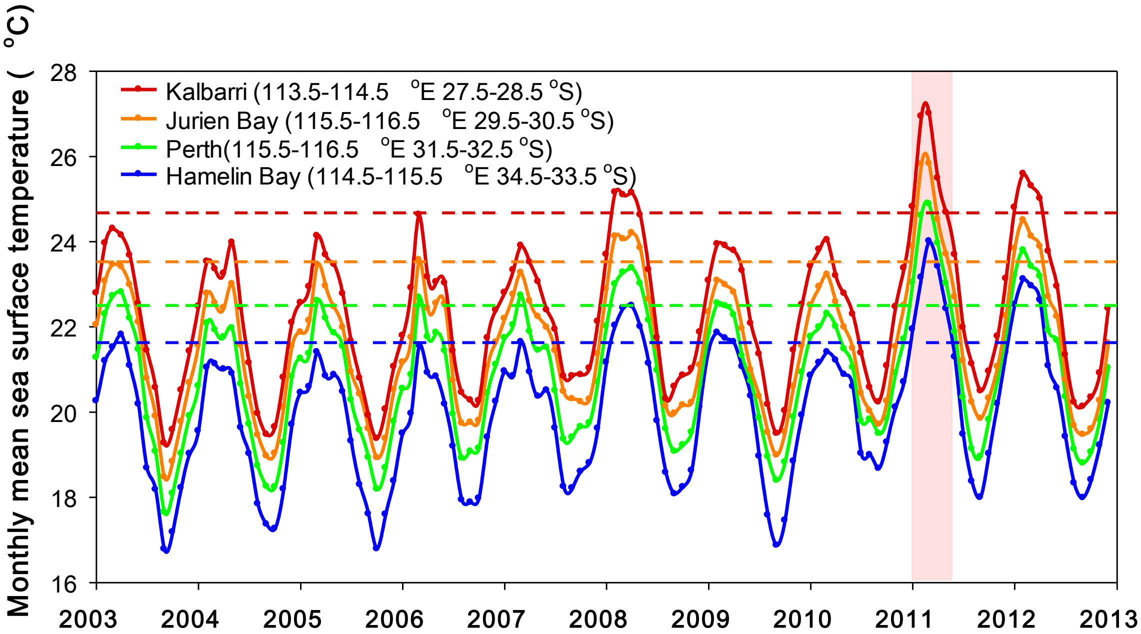

Fig. S1. *Ocean temperatures from January 2003 to December 2012. Monthly mean SST (solid lines) and maximum monthly mean temperatures for the climatological year 1981-2010 (broken lines). The five months (January to May 2011) encompassing the 2011 marine heatwave are highlighted in pale red.*

The heat wave peaked at slightly different times along the coast but warming profiles were similar in different regions (Fig. S1, Fig. S2). The satellites recorded marginally higher warming anomalies in Kalbarri (Fig. S2) but the highest absolute temperature anomalies have been reported from Jurien Bay (Pearce & Feng 2013), where kelp loss was only partial. Importantly, positive climatological maximum anomalies (CMA's) (i.e., conditions where ocean temperatures exceeded the climatological maximum temperature) prevailed for ~5 months throughout the coastline and there were no statistically significant differences between regions in CMA's over the duration of the heatwave (pair-wise t-tests, P > 0.213, Fig. S2).

Climatological maximum anomalies are more conservative, and provide a better measure of climatically stressful conditions, than simple temperature anomalies (Selig *et al.* 2010). In contrast to temperature anomalies, which provide the deviation from a fixed point in time, CMA's take into account that higher temperatures might normally occur at a different time of the year. That is, they report deviations from the maximum temperatures normally experienced at any time of the year (Selig *et al.* 2010). Here we report monthly CMA's as indicative of spatiotemporally broad patterns of heat exposure. Nevertheless, integrating and averaging inherently evens out short-term or localized extremes (Smale & Wernberg 2009), which might show different patterns that could also be important. Currently, there is a very limited understanding of what characteristics of a marine heatwave is detrimental to kelp forests and most other marine organisms (Hobday *et al.* 2016). Here we analyse cumulative climatological maximum anomalies because cumulative heat exposure (e.g., degree heating weeks) has proven to be a strong predictor for coral reefs (Selig *et al.* 2010) and this measure also takes into account that a heatwave can exhibit different characteristics in different locations (Hobday *et al.* 2016).

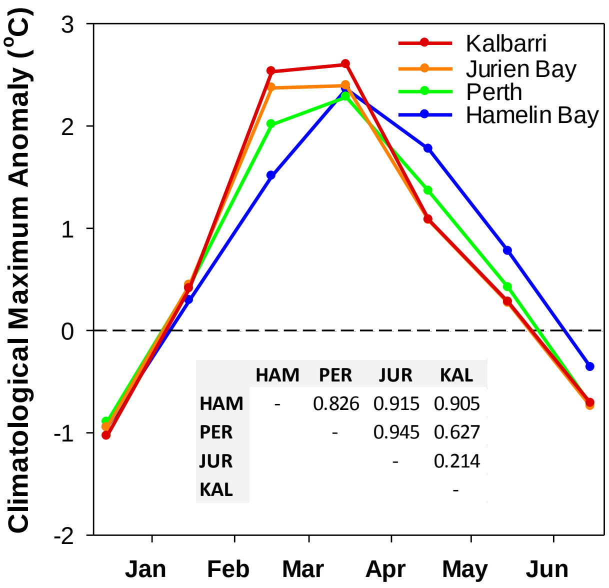

Fig. S2. *Climatological Maximum Anomalies (CMA's) for the five months encompassing the 2011-marine heat wave (CMA > 0; highlighted in red in Fig. S1) and two-tailed P-values (insert) from pair-wise comparisons of CMA's between all regions for the months January to May (CMA > 0).*

Appendix S3: Physical and biological predictor variables

Table S2. *Potential biological and environmental predictors of heatwave impacts. Data and their co-linearity are shown in Fig. S3.*

Predictor variable	Data description	Data source
Genetic diversity	Expected heterozygosity (H_e) derived from 6 polymorphic loci. Raw values used. Values assigned to each kelp forest by linear regression on latitude ($H_e = 0.0158 \times S° – 0.1643$; $r^2 = 0.77$, $P = 0.0004$).	Coleman *et al.* (2011)
Reef topography	Counts of large (>2 m vertical relief) topographical features per 25 m transect. Raw values used for each reef.	Tuya *et al.* (2009)
Fish herbivores	Counts of herbivorous fishes (*Kyphosus* spp., *Odax* spp, *Parma* spp.) observed in 1500 m^2 transects over one year prior to the 2011 marine heatwave. Raw values used for each reef.	Tuya *et al.* (2011)
Heatwave	Cumulative Climatological Maximum Anomaly. Sum of monthly temperatures exceeding the long-term climatological maximum (1981-2010) for each region (in Fig. S2, the area under each curve above zero). Akin to degrees heating weeks as it reports cumulative heat exposure. Kelp forests within a region were assigned identical CCMA's as short-term logger data has shown reef-to-reef differences to be less than 5% (Smale & Wernberg, 2009).	Appendix S2.
Wave exposure	Water motion at each kelp forest ranked from 1-12 based on many years of diving in all kelp forests.	Wernberg, unpublished data
Turf/foliose seaweeds	Measurements of cover of small turf and foliose algae cover along 25 m transects. Raw values used for each kelp forest.	Wernberg *et al.* (2011b)
Nutrient concentration	Mean NO_x concentration measured in each kelp forest 3 times over ~1 year. Raw values used.	Wernberg et al. (2010)
Depth	Measurements with a scuba depth gauge. Raw values used for each kelp forest.	Wernberg, unpublished data; see also Wernberg *et al.* (2010)

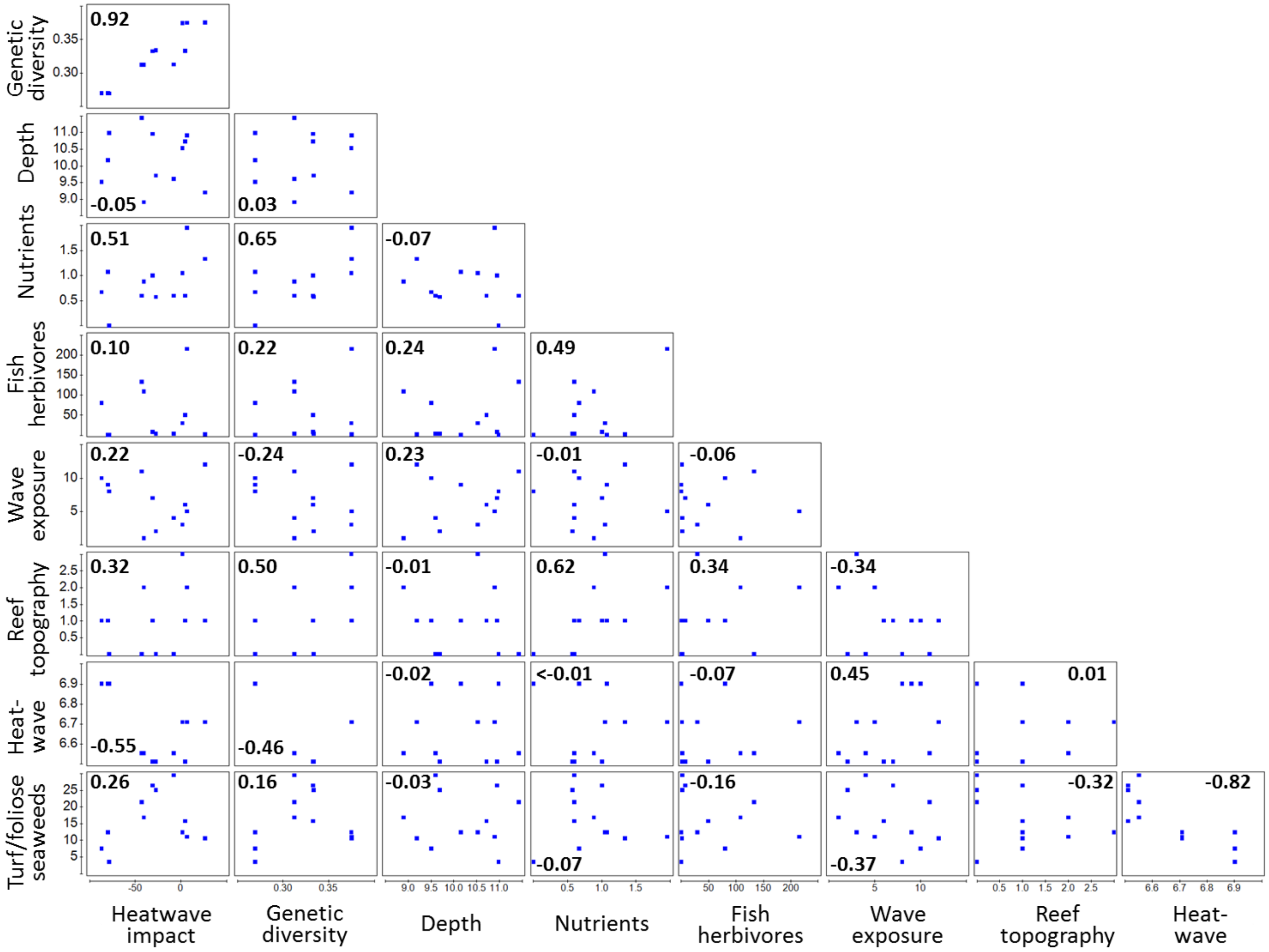

Fig. S3. *Draftmans plot of potential biological and environmental predictors of heatwave impacts (dependent variable) (cf. Table S2). Insert in each panel are Pearson's correlation coefficients (r) between each pair of biological and environmental predictor variables, as well as the dependent variable, heawwave impact, indicating their degree of co-linearity. Contributions from individual predictors that are highly correlated should be interpreted with caution.*

Appendix S4 Conceptual models

We speculate that two conceptual models (a 'subset' and an 'selection' model) may explain possible relationships between genetic diversity and population impacts of perturbations in these kelp forests (Fig. S4). Both models assume that individual genotypes vary in their stress tolerance, broadly defined as their capacity to resist and/or recover from a perturbation, and that their frequency of occurrence in the population follows a Gaussian distribution. Under the ‘subset model’, genetic diversity arises through non-selective processes (Hampe & Petit, 2005; Coleman et al., 2011), such that populations with low genetic diversity represent a narrow subset of genotypes compared to high diversity populations, while the genotypic population mean stress tolerance remains constant (Fig. S4a, b). Encountering increasingly stressful environmental conditions, low diversity populations transition from high (due to the absence of vulnerable genotypes) to low persistence as the environment exceeds the tolerance limits of intermediate genotypes (Fig. S4b, c). In contrast, diverse populations undergo more gradual decline in population persistence, due to the presence of both vulnerable and stress-tolerant genotypes. Under the ‘selection model’, low genetic diversity arises through selection of stress tolerant genotypes (Frankham, 2005; Hampe & Petit, 2005), such that genotypic mean population tolerance increases with loss of diversity (Fig. S4d, e). Consequently, under all but the most benign or extreme conditions, the ‘selection model’ predicts greater population resistance to perturbation and higher persistence of low relative to high diversity populations (Fig. 4f), whereas the opposite is true for the ‘subset model’ (Fig. S4c). In both cases, however, the response of low-diversity populations will be abrupt and catastrophic once the population threshold is exceeded (Fig. S4c, f).

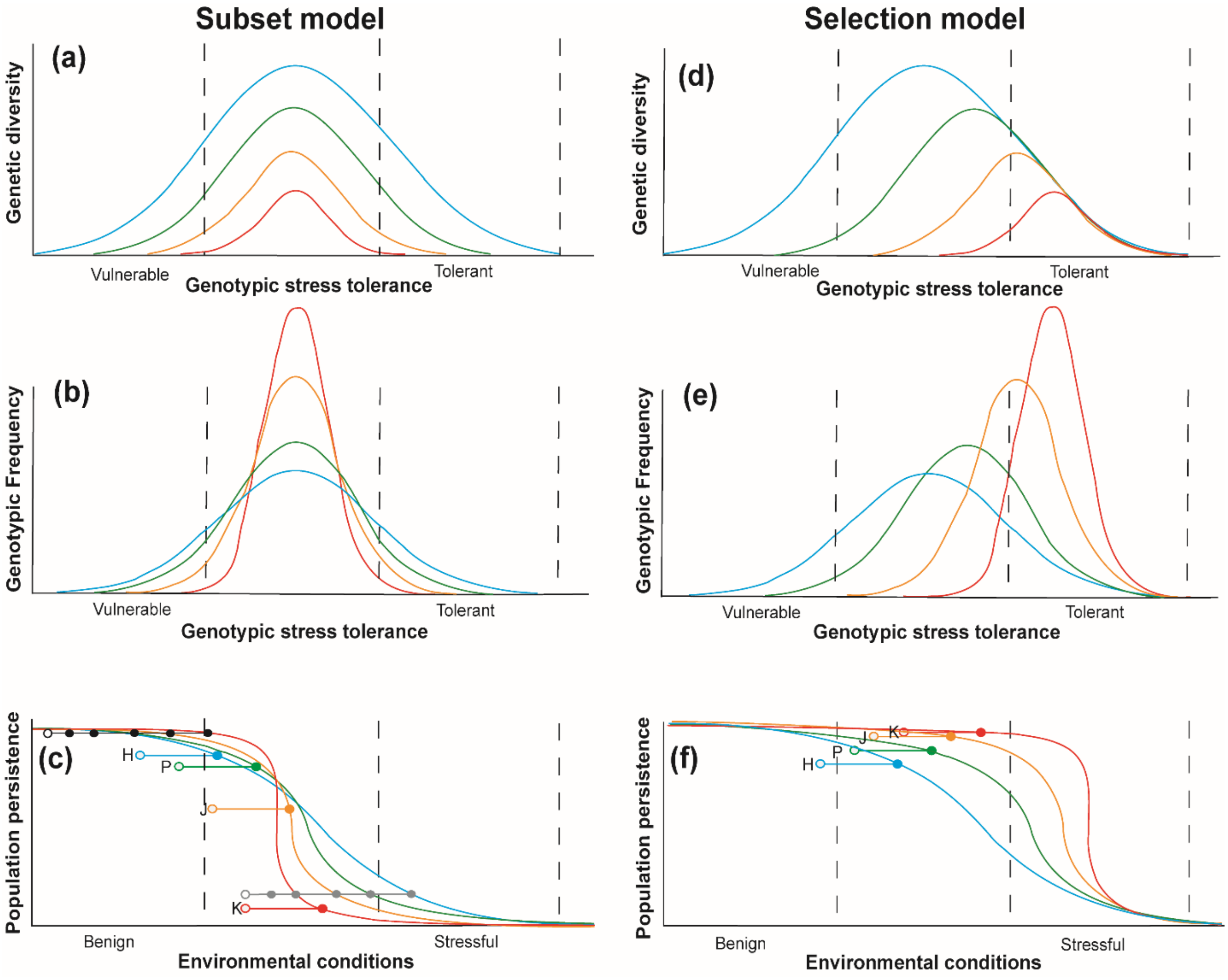

Figure S4. *Conceptual models of effects of genetic diversity on population responses to perturbation along an environmental gradient. Theoretical distribution of genetic diversity (a, d), population genotype frequencies (b, e) and population survival (c, f) under a 'subset' and an 'selection' origin of genetic diversity. In (c) and (f), different population endpoints (filled circles) are illustrated as a function of initial conditions (open circles) and magnitude of perturbation (length of the horizontal line). The colored examples (H, P, J, K) illustrate responses at our four regions (Hamelin Bay, Perth, Jurien Bay and Kalbarri, respectively), where genetic diversity and initial conditions differed (Fig. S1), but the magnitude of perturbation (monthly mean thermal stress anomaly) was similar (Fig. S2). The black and grey lines illustrate the general cases where variation in initial conditions, anomaly size and genetic diversity have no effect (black) or interact to produce complex patterns of influence on population survival (grey).*

Appendix S5 Additional references

Feng, M., McPhaden, M.J., Xie, S.-P., and Hafner, J. (2013) La Niña forces unprecedented Leeuwin Current warming in 2011. Scientific Reports 3, 1277

Pearce, A.F. and Feng, M. (2013). The rise and fall of the “marine heat wave” off Western Australia during the summer of 2010/2011. Journal of Marine Systems, 111–112, 139–156.

Selig, E.R., Casey, K.S., and Bruno, J.F. (2010) New insights into global patterns of ocean temperature anomalies: implications for coral reef health and management. Global Ecology and Biogeography 19, 397-411.

Smale, D.A. and Wernberg, T. (2009) Satellite-derived SST data as a proxy for water temperature in nearshore benthic ecology. Marine Ecology Progress Series 387:27-37

Tuya, F., Wernberg, T., and Thomsen, M.S. (2011) The relative influence of local to regional drivers of variation in reef fishes. Journal of Fish Biology 79, 217-234.

Zinke, J., Rountrey, A., Feng, M., Xie, S.-P., Dissard, D., Rankenburg, K. et al. (2014). Corals record long-term Leeuwin current variability including Ningaloo Nino/Nina since 1795. Nature Communications, 5, 3607.
